# Evaluation of the implementation of the objective structured clinical examination in health sciences education from a low‐income context in Tunisia: A cross‐sectional study

**DOI:** 10.1002/hsr2.2116

**Published:** 2024-05-13

**Authors:** Asma Ben Amor, Hassan Farhat, Guillaume Alinier, Amina Ounallah, Olfa Bouallegue

**Affiliations:** ^1^ Faculty of Medicine “Ibn El Jazzar” University of Sousse Sousse Tunisia; ^2^ Higher School of Health Sciences and Techniques University of Sousse Sousse Tunisia; ^3^ Ambulance Service Hamad Medical Corporation Doha Qatar; ^4^ Faculty of Sciences University of Sfax Sfax Tunisia; ^5^ School of Health and Social Work University of Hertfordshire Hatfield UK; ^6^ Weill Cornell Medicine‐Qatar Doha Qatar; ^7^ Faculty of Health and Life Sciences Northumbria University Newcastle upon Tyne UK; ^8^ Department of Dermatology Academic Hospital "Farhat Hached" Sousse Tunisia; ^9^ Microbiology Laboratory, Hygiene and Critical Care Departments Academic Hospital of Sahloul Sousse Tunisia

**Keywords:** health sciences, low‐resource settings, medical education, objective structured clinical examination, reliability

## Abstract

**Background:**

Objective structured clinical examination (OSCE) is well‐established and designed to evaluate students' clinical competence and practical skills in a standardized and objective manner. While OSCEs are widespread in higher‐income countries, their implementation in low‐resource settings presents unique challenges that warrant further investigation.

**Aim:**

This study aims to evaluate the perception of the health sciences students and their educators regarding deploying OSCEs within the School of Health Sciences and Techniques of Sousse (SHSTS) in Tunisia and their efficacity in healthcare education compared to traditional practical examination methods.

**Methods:**

This cross‐sectional study was conducted in June 2022, focusing on final‐year Health Sciences students at the SHSTS in Tunisia. The study participants were students and their educators involved in the OSCEs from June 6th to June 11th, 2022. Anonymous paper‐based 5‐point Likert scale satisfaction surveys were distributed to the students and their educators, with a separate set of questions for each. Spearman, Mann–Whitney *U* and Krusakll–Wallis tests were utilized to test the differences in satisfaction with the OSCEs among the students and educators. The Wilcoxon Rank test was utilized to examine the differences in students' assessment scores in the OSCEs and the traditional practical examination methods.

**Results:**

The satisfaction scores were high among health sciences educators and above average for students, with means of 3.82 ± 1.29 and 3.15 ± 0.56, respectively. The bivariate and multivariate analyzes indicated a significant difference in the satisfaction between the students' specialities. Further, a significant difference in their assessment scores distribution in the practical examinations and OSCEs was also demonstrated, with better performance in the OSCEs.

**Conclusion:**

Our study provides evidence of the relatively high level of satisfaction with the OSCEs and better performance compared to the traditional practical examinations. These findings advocate for the efficacy of OSCEs in low‐income countries and the need to sustain them.

## 1 INTRODUCTION

Assessing student learning capabilities is crucial to improving healthcare education by measuring students' knowledge and skills and contributing to their learning through instructional strategies. In recent years, a growing recognition has emerged regarding the need to align assessment practices with the evolving demands of healthcare education, which increasingly emphasizes the development of practical skills and competencies alongside theoretical knowledge.^1^, ^2^ Such alignment supports the integrity of academic accreditation and elevates the quality of education. This recognition suggests the need for a judicious selection of assessment strategies. Consequently, educators ensure a range of evaluation approaches that accurately measure students' cognitive capabilities and, as importantly, psychomotor and other professional skills.

For several decades, the objective structured clinical examination (OSCE) has emerged as a gold standard for appraising medical students, particularly as they conclude their clinical rotations.^3^ The OSCE is generally characterized by its rigorous methodology, reliability, and validity.^4^ This examination approach helps bridge the gap between theoretical learning and practical application, offering a simulated environment to evaluate multifaceted clinical competencies.^5^ The OSCE thereby fosters a shift from rote memorization to acquiring hands‐on skills indispensable for proficient healthcare delivery. This assessment method has gained popularity across multiple healthcare disciplines, including dentistry, physiotherapy, pharmacy, and engineering education.^6^, ^7^, ^8^, ^9^

The COVID‐19 pandemic has created various limitations in conducting practical assessments in medical education, leading health sciences educators worldwide to recalibrate their traditional educational methods. In response, Tunisia's Ministry of Higher Education has been at the forefront in catalyzing a paradigm shift towards more flexible and resilient educational strategies, including incorporating online simulations in OSCEs for the first time in Tunisia.^10^

In Tunisia, the medical sciences education is organized under the License‐Master‐Doctorate (LMD) system. This system covers various medical education specialities, such as nursing. It also covers various health sciences educational programs, including Emergency Medical Care (EMC), Anesthesia Technologists (AT), Radiology Technologists (RT), Biology Technologists (BT), Surgical Technologists (ST), Paediatric Care (PC) and podologists, among other specialities.^11^, ^12^ However, the LMD has not yet been implemented in medicine, dentistry, and pharmacy.^11^, ^12^ In Tunisia, the students allowed to continue in health sciences educational specialities programs are those who have successfully passed the national baccalaureate exam in experimental sciences or mathematics branches with respectable marks. These programs provide opportunities for capable students who could not secure admission or chose alternative paths beyond medicine, dentistry, or pharmacy programs yet still show a solid academic aptitude in their fields. In the Tunisian governmental medical education system, there are four faculties of medicine for medicine education, one faculty of dentistry for dentists, one faculty of pharmacy for pharmacists, four nursing institutes for nurses education and four Schools of Health Sciences and Technologies (SHST) for health sciences education. Each SHST is located near the biggest academic hospitals in Sousse, Monastir, Sfax and Tunis. They cover all the health sciences educational specialities (EMC, ST, RT, BT, and PC, among others).

Given the transformative trajectories that medical education will likely undertake in the post‐pandemic era, forward‐looking strategies are exigent. In the same context, OSCEs have recently been introduced into Tunisia's health sciences curricula. Hence, the perception of health sciences students and educators has never been measured. While OSCEs are universally recognized, little empirical inquiry exists to examine the feedback from health sciences students and educators from low‐resource settings like Tunisia. Although previous studies in Tunisia have evaluated the use of OSCEs in specific domains, such as medical internships for medical students,^10^, ^13^ an assessment of its application across multiple health sciences educational programs has been lacking. This study contributes to the limited literature by evaluating the adoption of OSCEs in health sciences education at the SHSTS of the University of Sousse—a low‐resource setting where constraints and educational traditions may pose unique challenges.^14^ By examining the perceptions and experiences of students and educators across various health sciences specialities, the research offers insights into implementing and accepting OSCEs within Tunisian health sciences education in other institutions. The multidisciplinary approach provides a broader perspective on the feasibility and potential barriers to integrating OSCEs across different health sciences programs in resource‐limited environments. This study aims to evaluate the health sciences students' and healthcare educators' perception of deploying OSCEs within the SHST of Sousse (SHSTS) at the University of Sousse in Tunisia and its perceived efficacity in medical education compared to the traditional practical examination methods.

## 2 METHODS

This cross‐sectional study was conducted in June 2022 for the SHSTS final‐year students in Sousse who had undergone the OSCE and the classic practical examinations in May and June 2022. The health sciences educational specialities in the SHSTS are EMC, ST, PC and Podology. The article's structure adopts the Consolidated Standards of Reporting Trials (CONSORT) checklist. Ethical approval for this study was obtained from the Faculty of Medicine “Ibn Eljazzar” Doctoral School of the University of Sousse review board on 27/03/2021.

### 2.1 Study design and setting

In the SHSTS, the OSCE is implemented as a comprehensive 1‐day examination. It is designed to evaluate health sciences students' skills and clinical capabilities in a rigorous, structured, reliable, and valid manner. In the SHSTS, the OSCE comprised five to seven stations, according to the students' specialities. Each station was run in a separate room for over 7 min. The students could read an instruction sheet on the door of each room, briefing them about the station's background and skill being assessed. An educator was in the room to assess the student's performance according to a pre‐defined evaluation grid without communicating any information. Once the student had finished performing the required task or reached 7 min without fully demonstrating the skill, the educator stopped the student and asked them to move quickly to the next station, as described in the summative OSCE approach in the article by Alinier.^5^ The evaluation grid included critical and non‐critical elements. Failure to perform one of the critical steps led to the student repeating the station another day after receiving feedback from the evaluator at the end of the OSCE examination day.

Anonymous paper‐based 5‐point Likert scale satisfaction surveys were distributed to the health sciences students and educators, with a separate set of questions for each (Appendixes 1 and 2). Participation in the study was entirely voluntary. Students were provided with a consent form that clearly stated they could choose whether or not to participate without any consequences. Those who wished to participate signed the consent form. For those who declined, there were no repercussions. To protect the privacy and confidentiality of participants, all responses to the surveys were anonymised. This ensured that students could provide honest feedback without fear of potential repercussions. Both surveys (Annex 1 and 2) included 6 demographic questions, 17 questions about the particularity of the OSCE, 10 about the structure, 9 about the organization, and 5 about its efficiency (validity and reliability) in assessing the students' skills.

In addition, all health sciences students participating in the OSCE underwent a 2‐h practical exam 2 weeks after the OSCE assessment dates. This exam involved practising a care skill in a real‐world scenario, where students interacted with actual patients and healthcare personnel in relevant departments, complementing the structured assessment of the OSCE. An educator was present during the practical exam to observe the students. While they did not interfere with the student's interactions, they assessed performance using a predefined checklist for each care task. The care tasks assigned to each student were randomly determined based on the clinical presentation of the patient and the student's health science educational specialities (e.g., Arterial blood gas test, Supra glottic and endotracheal airway control for EMC, and assisting in surgery by passing tools and retracting tissues for ST).

### 2.2 Participants and sampling

The study included the third‐year SHSTS all specialities students (the graduation year) (*N* = 133) and their educators (*N* = 33). Slovin's formula was utilized to determine the minimum sample size required: 98 for the students and 31 for the educators.

### 2.3 Data analysis

IBM‐SPSS version 26 was utilized for data analyzes. First, both surveys were validated using the Aiken V content validity coefficient (CVC) to determine whether the surveys measured what they intended to measure.^15^ Five experts in medical research and OSCE training were invited. A letter (Annex 3) explaining the study's objective was sent to them. These experts were asked to rate each survey item on a scale from 1 (lowest) to 5 (highest) for pertinence, clarity, and how well the item served as a good indicator of the intended measure. Then, based on their scoring, the CVC was calculated. Second, Cronbach alpha for reliability analysis was also determined. It aimed to determine whether or not we might get the same results if the surveys were repeated on another population with the same characteristics and under the same conditions. Third, descriptive statistics were conducted. The average of the satisfaction scores for each item was determined. Fourth, bivariate and multivariate analyzes were performed. The quantitative variables' Gaussian distribution was verified using Shapiro and Kolmogorov test. Then, accordingly, the Spearman tests were conducted to test the scores' correlation between the variables. The Man–Whitney *U* test was performed to test the distribution of the satisfaction score within each health sciences student group.

Furthermore, the Wilcoxon signed ranks test was utilized to evaluate whether there were statistically significant differences between the scores of traditional practical examinations and the average score of the health sciences students in the various sections. The Kruskal–Wallis test was performed to test the following hypotheses: Then, based on the Kruskal–Wallis test results, the post hoc test was conducted to determine which samples had different satisfaction distributions. Shewhart chart was performed to analyze the variation of the satisfaction scores across the group.

## 3 RESULTS

A total of 131 students and 33 educators participated in the surveys. Table 1 presents demographic information of the participating population.

**Table 1:** Demographic information.

**Health sciences students**		Age	
		Mean	22
		Standard deviation	1
		Median	21
		Percentile 05	21
		Percentile 25	22
		Percentile 75	23
		Percentile 95	47
	Categorical variables	Number	Count
	Gender		
	Female	128	100%
	Educational programs		
	Podology	20	11.40%
	Emergency medical care	35	20%
	Surgical technologists	34	19.40%
	Paediatric care	39	22.3%
**Health sciences educators**		Age	
		Mean	41
		Median	40
		Standard deviation	10
		Percentile 05	27
		Percentile 25	35
		Percentile 75	43
		Percentile 95	61
		Experience	
		Mean	13
		Median	
		Standard deviation	11
		Percentile 05	4
		Percentile 25	6
		Percentile 75	14
		Percentile 95	38
	Categorical variables	Number	Count
	Background		
	Nurses	6	3.41%
	Health sciences professors	12	6.90%
	Senior health sciences technologist	13	7.42%
	Highest academic degree		
	Master of research	11	6.34%
	Master of sciences	1	0.61%
	Other	12	6.92%
	Professors of paramedical education	5	2.91%
	PhD	2	1.17%
	Classes teaching		
	Podology	6	3.40%
	Emergency medical care	13	7.47%
	Surgical technologists	7	4.01%
	Paediatric care	5	2.91%

The CVCs of the health sciences students and educators' surveys were determined. They were respectively equal to 0.71 ± 0.44 (Table 2) and 0.82 ± 0.02 (Table 3), indicating a solid validity of both tools. The reliability of both tools was also assessed in Table 4, giving Cronbach alpha coefficients for both tools equal to 0.96 and 0.83, indicating the solid reliability of both tools and that they would give the same results if repeated under the same circumstances.

**Table 2:** Students' Aiken V validity coefficient results.

Overall results										
Pertinence			Clarity		Good indicator		Overall		Confidence interval (95%)	
0.74			0.69		0.70		0.71		±0.45	

Detailed results										
**Items**	**Pertinence**	**Clarity**	**Good indicator**	**Overall**	**Items**	**Pertinence**	**Clarity**	**Good indicator**		**Overall**
**Q1**	1	1	1	1	**Q22**	0.75	0.71	0.75		0.73
**Q2**	1	1	1	1	**Q23**	0.60	0.71	0.60		0.64
**Q3**	1	1	1	1	**Q24**	0.60	0.64	0.60		0.61
**Q4**	1	1	1	1	**Q25**	0.71	0.71	0.71		0.71
**Q5**	0.78	0.7	0.78	0.76	**Q26**	0.53	0.53	0.53		0.53
**Q6**	0.67	0.82	0.67	0.72	**Q27**	0.35	0.35	0.35		0.35
**Q7**	0.71	0.67	0.71	0.70	**Q28**	0.60	0.64	0.60		0.61
**Q8**	0.85	0.75	0.85	0.82	**Q29**	0.50	0.46	0.50		0.48
**Q9**	0.82	0.75	0.82	0.79	**Q30**	0.82	0.78	0.82		0.81
**Q10**	0.78	0.71	0.78	0.76	**Q31**	0.71	0.64	0.71		0.69
**Q11**	0.85	0.89	0.85	0.86	**Q32**	0.64	0.67	0.64		0.65
**Q12**	0.82	0.82	0.82	0.82	**Q33**	0.57	0.57	0.57		0.57
**Q13**	0.78	0.78	0.78	0.78	**Q34**	0.67	0.67	0.67		0.67
**Q14**	0.67	0.67	0.67	0.67	**Q35**	0.71	0.64	0.71		0.69
**Q15**	0.67	0.67	0.67	0.67	**Q36**	0.89	0.67	0.89		0.82
**Q16**	0.67	0.67	0.67	0.67	**Q37**	0.89	0.92	0.89		0.90
**Q17**	0.60	0.67	0.60	0.63	**Q38**	0.96	0.96	0.96		0.96
**Q18**	0.71	0.78	0.71	0.73	**Q39**	0.92	0.92	0.92		0.92
**Q19**	0.71	0.71	0.71	0.71	**Q40**	0.85	0.85	0.85		0.85
**Q20**	0.57	0.64	0.57	0.59	**Q41**	0.60	0.53	0.60		0.58
**Q21**	0.78	0.71	0.78	0.76	**Q42**	0.71	0.75	0.75		0.73

**Table 3:** Health sciences educators'Aiken V validity coefficient results.

Overall results										
Pertinence		Clarity		Good indicator		Overall		Confidence interval (95%)		
0.82		0.82		0.82		0.82		±0.03		

Detailed results										
**Questions**	**Pertinence**	**Clarity**	**Good indicator**	**Overall**	**Questions**	**Pertinence**	**Clarity**		**Good indicator**	**Overall**
**Q1**	1	1	1	1	**Q26**	0.89	0.86		0.89	0.88
**Q2**	1	1	1	1	**Q27**	0.86	0.89		0.86	0.87
**Q3**	0.75	0.75	0.75	0.75	**Q28**	0.75	0.71		0.75	0.74
**Q4**	0.82	0.82	0.82	0.82	**Q29**	0.79	0.75		0.79	0.77
**Q5**	0.75	0.75	0.75	0.75	**Q30**	0.79	0.82		0.79	0.80
**Q6**	0.71	0.71	0.71	0.71	**Q31**	0.79	0.75		0.79	0.78
**Q7**	0.89	0.86	0.89	0.88	**Q32**	0.79	0.79		0.79	0.79
**Q8**	0.86	0.86	0.86	0.86	**Q33**	0.75	0.75		0.75	0.75
**Q9**	0.86	0.89	0.86	0.87	**Q34**	0.86	0.82		0.86	0.85
**Q10**	0.71	0.75	0.71	0.73	**Q35**	0.82	0.82		0.82	0.82
**Q11**	0.75	0.82	0.75	0.78	**Q36**	0.93	0.93		0.93	0.93
**Q12**	0.64	0.64	0.64	0.64	**Q37**	0.93	0.82		0.93	0.89
**Q13**	0.75	0.71	0.75	0.74	**Q38**	0.75	0.71		0.75	0.74
**Q14**	0.89	0.82	0.89	0.87	**Q39**	0.82	0.82		0.82	0.82
**Q15**	0.68	0.71	0.68	0.70	**Q40**	0.82	0.79		0.82	0.810
**Q16**	0.79	0.79	0.79	0.79	**Q41**	0.89	0.79		0.89	0.86
**Q17**	0.75	0.86	0.75	0.79	**Q42**	0.82	0.93		0.82	0.86
**Q18**	0.71	0.75	0.71	0.73	**Q43**	0.96	0.89		0.89	0.92
**Q19**	0.71	0.79	0.71	0.74	**Q44**	0.93	0.964		0.79	0.89
**Q20**	0.71	0.71	0.71	0.71	**Q45**	0.82	0.964		0.96	0.92
**Q21**	0.68	0.71	0.68	0.69	**Q46**	0.96	0.79		0.96	0.90
**Q22**	0.71	0.71	0.71	0.71	**Q47**	0.93	0.96		1	0.96
**Q23**	0.86	0.89	0.86	0.87	**Q48**	0.96	0.96		1	0.98
**Q24**	0.89	0.93	0.89	0.90	**Q49**	0.93	0.86		0.96	0.92
**Q25**	0.82	0.82	0.82	0.82						

**Table 4:** Health sciences students and educators' surveys reliability statistics.

Students survey reliability statistics		
Cronbach alpha	Cronbach alpha based on standardized items	Number of items
0.96	0.96	38

Health sciences educators' survey reliability statistics		
Cronbach alpha	Cronbach alpha based on standardized items	Number of items
0.81	0.84	44

**Figure 1:**
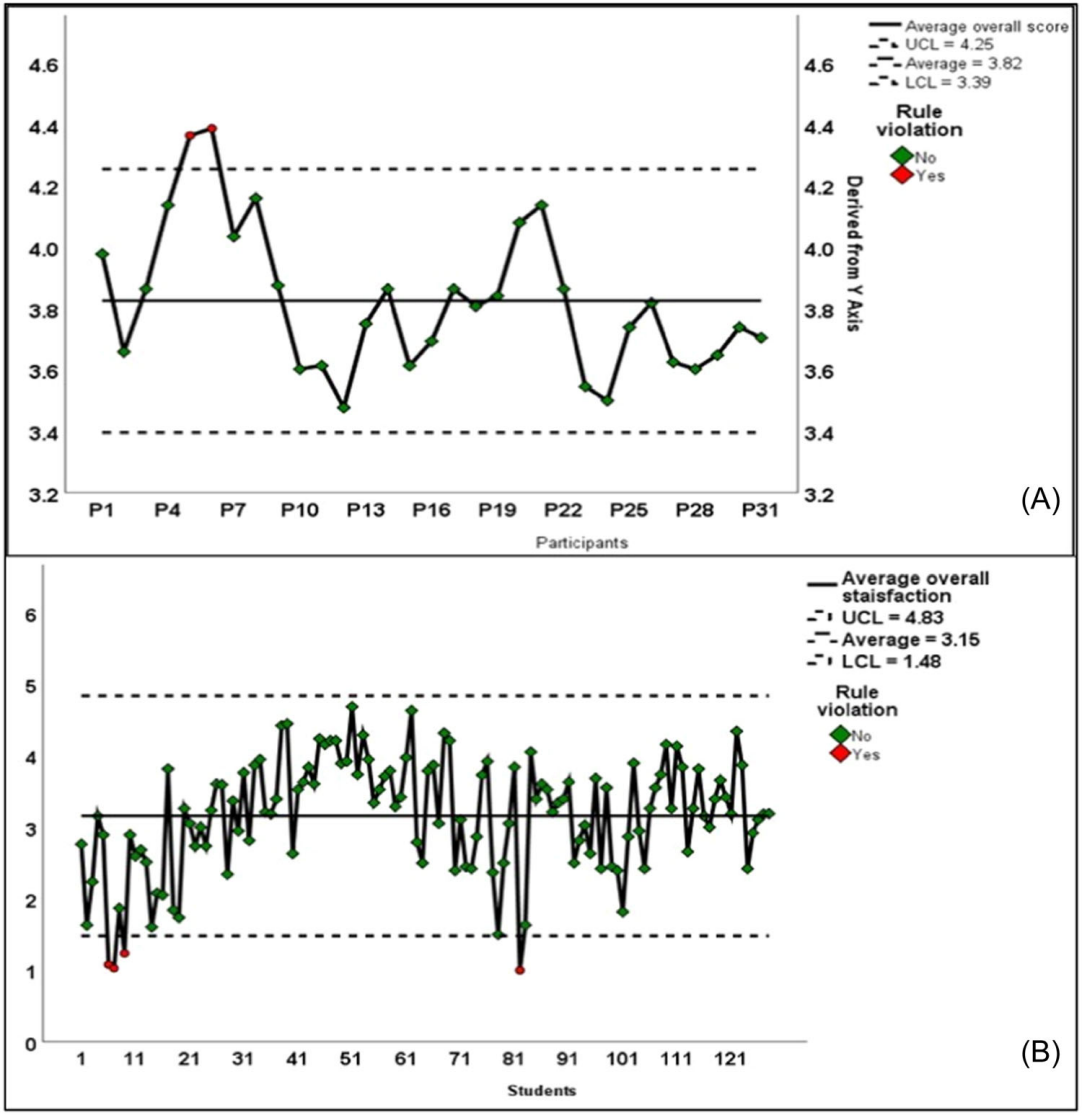
Shewhart chart for students' (B) and educators' (A) average satisfaction scores.

The average overall satisfaction scores of health sciences students and educators were determined and represented in the Shewhart charts in Figure 1. In Shewhart charts, the control limits are calculated based on the data and represent the natural boundaries within which the process is considered stable and desirable.^16^ The upper control limit (UCL) and lower control limit (LCL) are set at three standard deviations above and below the mean, respectively. In Figure 1A, the educators' satisfaction scores varied mostly within the control limits, indicating a stable process. Furthermore, the mean satisfaction score for educators was above 3, which is considered a high satisfaction level based on the 5‐point Likert scale used in the survey. In Figure 1B, the students' satisfaction scores varied mainly within the control limits, suggesting an overall stable process. However, the mean satisfaction score for students fluctuated between high (≥3) and low (<3) levels on the 5‐point Likert scale, indicating more variability in their satisfaction compared to the educators.

The quantitative variable distributions (students' ages, health sciences educators' ages, years of experience, and satisfaction scores) were verified using the Shapiro test. All variables' *p*‐values were <0.05, indicating that these quantitative variables were not normally distributed. Table 3 indicates the bivariate and multivariate analysis results.

Further, in Table 5, for the health sciences educators, there was a fair, positive correlation between the particularity and structure of the OSCE and a strong positive correlation between the age and experience of the health sciences educators and the overall satisfaction scores. The Kruskall–Wallis and post hoc tests indicated a significant difference in the satisfaction distribution between the health sciences educators' groups according to their backgrounds. It is worth mentioning here that the health sciences educators' backgrounds were classified according to their specialities before they underwent the pathway of health sciences education. The post hoc test indicated the highest satisfaction among educators who previously studied EMC and ST.

**Table 5:** Bivariate and multivariate analysis results for the Health sciences educators.

	Particularity^a^	Structure^a^	Organization^a^	Efficiency^a^	Overall satisfaction score^b^
Particularity^a^	1	0.44 (0.01)	0.27 (0.15)	0.16 (0.39)	0.69 (0.00)
Structure		1	0.10 (0.57)	0.27 (0.14)	0.389 (0.03)
Organization			1	0.39 (0.03)	0.684 (0.00)
Efficiency				1	0.604 (0.00)
Overall satisfaction score	Gender^c^	Age^b^	Experience^b^	Sections^d^	Background
	0.29	0.91 (0.00)	0.91 (0.00)	0.77	0.09^e^
	Highest academic degree^d^	Structure	Particularity	Organization	Efficiency
	>0.05	>0.05	>0.05	>0.05	>0.05

^a^ Pearson correlation.

^b^ Spearman correlation (Rho).

^c^ Mann–Whitney *U* test.

^d^ Kruskal–Wallis Test.

^e^ Post hoc.

Additionally, in Table 6, for the students, there is a strong positive correlation between the students' satisfaction variables. Kruskall–Wallis and post hoc tests demonstrated that EMC students were more satisfied than the ST, followed by PC and podology students.

**Table 6:** Bivariate and multivariate analysis results for the senior health technology students.

	Particularity^a^	Structure^a^	Organization^a^	Efficiency^a^	Overall satisfaction score^a^
Particularity^a^	1	0.44 (0.01)	0.57 (0.00)	0.78 (0.00)	0.82 (0.00)
Structure		1	0.59 (0.00)	0.63 (0.14)	0.39 (0.03)
Organization			1	0.62 (0.00)	0.82 (0.000)
Efficiency				1	0.90 (0.00)
Overall satisfaction score	Age^b^	Sections^c^	Particularity		
	>0.05	0.000^d^	>0.05		
	Structure	Organization	Efficiency		
	>0.05	>0.05	>0.05		

^a^ Spearman correlation (Rho).

^b^ Mann–Whitney *U* test.

^c^ Kruskal–Wallis test.

^d^ Post hoc.

Finally, the results in Table 7 indicate significant differences in scores between the practical examinations and OSCE in the EMC, ST, and PC sections, with OSCE scores tending to be higher. No significant difference was found in the podologists section. The descriptive statistics provide additional insights into the average scores and their distribution within each section.

**Table 7:** The descriptive and Wilcoxon Rank test for the practical and OSCE tests' results.

Descriptive statistics									
						Percentiles			
Section	*N*	Mean	Standard deviation	Min	Max	25th	50th (Median)	75th	
Emergency medical care								
Practical	36	15.57	2.92	0.00	17.75	14.75	16.13	17.00	
OSCE	36	16.91	1.11	13.65	18.47	16.33	16.87	17.76	
Surgical technologists									
Practical	36	13.91	5.87	0.00	18.50	14.50	15.88	17.50	
OSCE	36	13.37	2.82	0.00	16.43	12.22	13.53	15.01	
Paediatric care								
Practical	38	14.12	1.67	8.00	16.75	13.19	14.19	15.07	
OSCE	38	14.81	0.97	13.00	17.20	14	14.75	15.48	
Podology									
Practical	20	13.51	4.73	0.00	17.75	14.16	14.50	15.66	
OSCE	20	13.81	1.60	10.05	17.05	12.83	13.82	15	
Wilcoxon signed ranks test									
Section			Z	Asymp. Sig. (two‐tailed) (OSCE stations vs. practicial exam)					
Emergency medical care			−3.65^a^	0.00					
Surgical technologists			−2.54^b^	0.011					
Paediatric care			−2.36^a^	0.018					
Podology			−1.31^b^	0.19					

^a^ Based on negative ranks.

^b^ Based on positive ranks.

## 4 DISCUSSION

In a controlled and reproducible environment, OSCEs offer a standardized, objective, and comprehensive approach to assessing clinical skills, including communication, history‐taking, physical examination, and clinical reasoning. This structured assessment method has gained significant traction in medical and allied health education globally, as it provides a more reliable and valid measure of clinical competence compared to traditional assessment methods, such as written examinations or unstructured clinical evaluations.

The present study identified various insights regarding the students' and educators' perception of OSCEs, particularly in a Tunisian low‐resource environment like the SHSTS. Our findings demonstrated the robust validity and reliability of OSCEs, echoing previous research that has established OSCEs as a reliable and positively appreciated assessment strategy in healthcare education.^3^, ^5^, ^17^, ^18^ This validation is crucial, especially in a Tunisian context where new methods are introduced, signaling a potential shift in healthcare education assessment within low‐resource settings. The demographic findings revealed that satisfaction scores were high among educators but showed more variance among students. This discrepancy in satisfaction rates could reflect a range of factors, including the novelty of OSCEs in Tunisia, varying expectations, and differing levels of familiarity and stress associated with this assessment format. One approach to consider is organizing formative OSCEs so educators and students can become more acquainted with this assessment approach. An additional way of dissipating the students' stress associated with potentially underperforming in a given station is to ensure that an OSCE is constituted of a higher number of stations, sometimes including “theoretical stations,” hence ensuring that each skill is assessed several times in different ways and contexts.^8^, ^19^ This also contributes to increasing the validity and reliability of the overall assessment approach.

Such a divergence in satisfaction rates between these two groups warrants further investigation into the underlying causes of the pedagogical backgrounds and training. They can profoundly influence satisfaction levels, a crucial metric in educational quality. This aligns with the traditional didactic method, characterized by lecturer‐led teaching. While this approach has long been considered essential, its efficacy in meeting contemporary educational needs in all environments is increasingly questioned.^20^ Studies show that didactic learning may need to improve in fostering critical thinking and practical skills while efficiently disseminating factual knowledge, potentially leading to lower satisfaction levels among students who request a more interactive and engaging learning environment.^17^, ^21^ In contrast, problem‐based learning, such as the OSCE, represents a suitable change, emphasizing student‐centered learning and practical problem‐solving skills.^22^ Problem‐based learning has been shown to enhance student satisfaction by actively engaging learners in the educational process, promoting more profound understanding, and fostering critical thinking. However, the success of problem‐based learning is contingent upon the health sciences educators' ability and willingness to facilitate rather than direct learning, which can be a significant cultural and pedagogical shift for faculty accustomed to traditional methods.^23^ Integrating simulation‐based training provides a safe, controlled environment for practising clinical skills,^19^ which, in our study, has been positively correlated with both student and educator satisfaction due to its practicality and relevance to clinical practice.

Moreover, the positive perceptions of Tunisian health sciences educators and students suggest a readiness and willingness to embrace this assessment approach, which could drive educational reform and quality improvement within the country's health sciences curricula. The divergence in satisfaction rates between these two groups warrants further investigation into the underlying factors, such as pedagogical backgrounds and training approaches. Addressing these disparities through targeted training and familiarization initiatives would further enhance the acceptance and effectiveness of OSCEs in Tunisian health sciences education. The successful implementation of OSCEs in Tunisia represents a groundbreaking move towards aligning the country's medical education with international best practices, particularly in resource‐limited settings. The need for quality improvement in healthcare education is pressing, and standardized, internationally recognized assessment methods like the OSCE would be crucial in driving educational reform. This change improves assessment practices and enhances educational objectives, ensuring that teaching methods and student performance evaluations are coherent, comprehensive, and conducive to producing competent healthcare professionals.

Introducing OSCEs in Tunisia represents a ground‐breaking move towards aligning the country's medical education with global standards, particularly in low‐resource contexts. The need for quality improvement in healthcare education is pressing, and standardized, internationally recognized assessment methods like the OSCE could be crucial in driving educational reform. This change improves assessment and enhances educational objectives, ensuring that teaching methods and student performance evaluations are coherent, comprehensive, and conducive to producing competent healthcare professionals.^24^ Globally, OSCEs are increasingly recognized for their utility in diverse educational contexts. In higher‐income countries like the United Kingdom and the United States, OSCEs are integral to medical and nursing education.^25^, ^26^ Their adoption in lower‐middle‐income countries, including India and Nigeria, indicates a growing acknowledgment of their effectiveness.^27^, ^28^

Furthermore, the findings presented in this study, which indicate that students achieved better scores in the OSCE compared to traditional practical assessments, highlight the potential benefits of this assessment approach in accurately evaluating clinical competencies. The controlled and standardized nature of OSCEs mitigates biases inherent in real‐world practical examinations, where factors such as patient conditions and reactions may distract students from focusing on the assessed skills. By providing a simulated yet realistic environment, OSCEs enable a more objective and focused evaluation of students' abilities, potentially leading to more accurate and reliable assessments of their readiness for clinical practice.

However, the introduction of OSCEs in new contexts like Tunisia is challenging. Institutional traditions, health sciences educators' capabilities, and students' familiarity are barriers that must be considered. Our study is a foundational step in this direction, providing a model that can be adapted and refined for broader implementation. Furthermore, the results presented in Table 7 indicate that most students achieved better scores in the OSCE compared to the traditional practical assessment. This latter method relies on direct interaction with patients to perform clinical skills pertinent to health sciences practice, with students receiving assistance from full‐time clinical personnel in the department. In such assessments, the health sciences educators must remain impartial. However, while offering real‐time feedback, this approach can be biased due to various factors, such as patient conditions and reactions. These may distract the students from focusing on the assessed skills, potentially impairing their performance. Moreover, in low‐income countries like Tunisia, where continuous education for health sciences personnel is not as well‐established as in countries such as Finland, the United Kingdom, and other countries,^29^, ^30^, ^31^ the regular staff's commitment to best practices might be compromised, consequently affecting students' performance and resulting in lower scores on the evaluation grid. In contrast, the OSCE method simulates a real‐life scenario while challenging the student to complete the skill within a designated timeframe, free from the biases inherent in a real practical examination. Therefore, using the OSCE as an evaluation method is recommended over the traditional practical examination format.

Further, high‐quality healthcare education is a global imperative, yet access to it in low‐income countries needs improvement in the delivery and assessment.^32^ Integrating reliable and valid assessment methodologies like the OSCE into settings such as Tunisia is significant. It represents a move towards global best practices in healthcare education, potentially driving quality improvement and boosting the pursuit of educational excellence and healthcare quality in the region across similar low‐income settings. Moreover, the COVID‐19 pandemic has highlighted the importance of adaptable, safe educational practices. The pandemic has highlighted the challenges faced by resource‐limited healthcare systems, emphasizing the need for robust healthcare education in such contexts.^33^, ^34^ The pandemic has also emphasized the importance of adaptable and resilient educational strategies, including incorporating online simulations and virtual OSCEs.^35^ As SHSTS at the University of Sousse, Tunisia, is a pioneer in implementing OSCEs in health sciences education within the country, SHSTS's proactive response in catalyzing a culture of resilient educational strategies during the COVID‐19 pandemic demonstrates a solid commitment to embracing innovative approaches and ensuring the continuity of high‐quality healthcare education despite the unprecedented challenges posed by the public health crisis. Our study's relatively high satisfaction levels showed that the OSCEs offer a controlled environment for clinical competence assessment.^36^, ^37^

In conclusion, our study corroborates the high levels of validity and reliability of OSCEs and survey instruments, aligning with existing research. The relatively high satisfaction levels reported among health sciences educators and students in the SHSTS, coupled with the demonstrated efficacy of OSCEs in accurately assessing clinical competencies, highlight the potential for this assessment approach to drive transformative change in the health sciences education at the University of Sousse in Tunisia, contributes to the development of a more competent and well‐prepared healthcare workforce, capable of delivering high‐quality patient care in resource‐limited settings. It addresses a critical literature gap by setting its research in a low‐income country, providing empirical evidence of the need to adapt medical education techniques for resource‐limited settings.

## 5 LIMITATIONS

The study was unicentric, restricting the generalizability of the results. While robust, the reliability and validity indices may exhibit different characteristics in an expanded sample encompassing various medical centers with varying resources, curricula, pedagogical practices and a higher number of OSCE stations. A broader, multi‐institutional approach could provide a more comprehensive view and mitigate the potential for selection bias, thereby enhancing the external validity of the findings.

Furthermore, while the findings explored the perception of OSCEs within the Tunisian medical education context, a more extended examination period could yield additional insights and enhance the reliability of the results. Future research should consider longitudinal studies spanning multiple weeks or months to fully understand the long‐term impacts, challenges, and adaptations associated with integrating OSCEs into the curriculum. Prolonged observation periods would allow researchers to assess the sustainability of the observed satisfaction levels among students and educators and monitor potential shifts in perceptions and performance over time.

## 6 CONCLUSION

While the study provides evidence for the efficacy of OSCEs in evaluating students' performance and instructors' evaluation of this assessment approach, it also highlights the need for further research to substantiate and expand upon these findings. Future studies could explore alternative statistical frameworks for enhanced analytical insights and extend the research to a more diverse range of medical centers for increased generalizability. Introducing OSCEs into Tunisian healthcare education is a pioneering initiative with potential ramifications for improving healthcare education in Tunisia and similar low‐resource environments globally.

## AUTHOR CONTRIBUTIONS

**Asma Ben Amor**: Writing — original draft; methodology; conceptualization; investigation. **Hassan Farhat**: Writing — original draft; methodology; data curation; visualization; formal analysis; validation; investigation. **Guillaume Alinier**: Writing — review & editing. **Amina Ounallah**: Project administration; supervision; Writing — review & editing. **Olfa Bouallegue**: Writing — review & editing; project administration; supervision. All authors read the manuscripts and approved the content for publication.

## CONFLICT OF INTEREST STATEMENT

The authors declare no conflicts of interest.

## ETHICS STATEMENT

This study was approved by the director of the doctoral school in the faculty of medicine of Sousse and by the management team of the School of Health Sciences and Medical Technologies of Sousse on 27/03/2021.

## TRANSPARENCY STATEMENT

The lead author Hassan Farhat affirms that this manuscript is an honest, accurate, and transparent account of the study being reported; that no important aspects of the study have been omitted; and that any discrepancies from the study as planned (and, if relevant, registered) have been explained.

## Data Availability

The data that support the findings of this study are available on request from the corresponding author. The data are not publicly available due to privacy or ethical restrictions.

## APPENDIX 1 HEALTH SCIENCES EDUCATORS SURVEY QUESTIONS (AS CIRCULATED IN FRENCH).

**Table:**

Caractéristiques sociodémographiques						
1. Age						
2. Genre MAS □ FEM □						
3. Ancienneté						
Section: Urgence &rea □ instrumentation. Op □ S.périculture □ podologie □						
4. Grade:						
5. Dernier diplôme obtenu:						

**Items**	**Tout à fait d'accord**		**D'accord**	**incertain**	**Pas d'accord**	**Pas de tout d'accord**
**caractéristiques de L'ECOS**						
6. Cette forme d'évaluation vous semble applicable et utile dans le programme LMD en santé						
7. L'ECOS couvre un large éventail de compétences et de connaissances cliniques						
8. L'ECOS doit être sommative et formative						
9. L'ECOS réduit les chances d'échec pour l'étudiant						
10. L'ECOS aide les étudiants à acquérir de la confiance tout en pratiquant						
11. L'ECOS aide le personnel enseignant à évaluer son propre niveau de connaissances						
12. L'ECOS aide le personnel enseignant à évaluer leurs propres aptitudes psychomotrices						
13. Je peux préparer et utilizer l'ECOS						
14. L'élaboration des stations est facile à réaliser						
15. L'ECOS prend beaucoup de temps pour la préparation par rapport à la clinique normative classique « sur le terrain »						
**Structure de L'ECOS**						
16. Le format de notation de l'ECOS était approprié pour évaluer les étudiants sur des stations particulières						
17. L'information en amont sur le déroulement de l'épreuve vous a semblé suffisante						
18. Les instructions aux élèves à chaque station étaient claires et sans ambiguïté						
19. L'épreuve vous a semblé proche de la réalité (stage sur terrain)						
20. le temps de participation à cette épreuve vous a semblé Beaucoup plus long Pour l'étudiant par rapport à l'examen clinique						
21. le temps de participation à cette épreuve vous a semblé Beaucoup plus long Pour l'enseignant, par rapport à l'examen clinique						
22. La séquence des stations était logique et appropriée						
23. Le temps alloué à chaque station était suffisant						
24. Le nombre de stations était suffisant						
25. Les stations et les paramètres reflétaient un scénario clinique authentique						
**Organization de L'ECOS**						
26. L'ECOS prend beaucoup de temps pour la préparation par rapport à la clinique normative classique « sur le terrain »						
27. Elaboration de la grille d'évaluation est facile à réaliser						
28. La gestion du matériel est facile à réaliser						
29. La gestion des locaux est facile à réaliser						
30. La gestion du parcours est facile à réaliser						
31. La gestions de personnel évaluateur est facile à réaliser						
32. La gestion des acteurs (patient simulé) est facile à réaliser						
33. L'examen était bien organisé et bien administré						
34. Les équipements comprenant des simulateurs, des instruments médicaux et le matériel nécessaire étaient disponibles et étaient de bonne qualité						
**Validité et fiabilité de l'ECOS**						
35. Les résultats de l'ECOS fournissent une véritable mesure des compétences cliniques essentielles						
36. L'ECOS a un impact positif sur l'apprentissage des étudiants						
37. L'examen était stressant pour les étudiants						
38. L'ECOS est un examen standardisé pour tous les étudiants						
39. L'ECOS est clair et impartial						
40. L'ECOS est équitable pour tous les étudiants						
41. L'ECOS évalue tous les étudiants objectivement						
42. les notes obtenues par les participants sont en adéquation avec la prestation						
43. L'ECOS devrait être utilisé plus souvent dans les autres années cliniques						
44. L'ECOS est préférable à d'autres formes d'examen clinique (examen du stage)						
45. Avoir des questions et des scénarios semblables pour tous les élèves est une bonne chose						
46. L'ECOS donne une rétroaction sur le rendement qui peut être utilisé pour l'auto‐amélioration						
47. L'ECOS reflète les exigences de la profession médicale						
48. La personnalité et le sexe n'affecteront pas les scores de L'ECOS						
49. Une formation pédagogique à l'égard de L'ECOS vous semble utile						

## APPENDIX 2 STUDENTS' SURVEY'S QUESTIONS.

**Table:**

Caractéristiques sociodémographiques							
1. Age							
2. Genre	MAS □				FEM □		
3. Niveau d'étude	1ere année □ 2ieme année□ 3ieme année□						
4. Section: Urgence &rea □ instrumentation. op □ S.périculture □ podologie □							

Items		**Tout à fait d'accord**	**D'accord**	**Incertain**		**Pas d'accord**	**Pas de tout d'accord**
**Structure de L'ECOS**							
5. L'information sur le déroulement de l'épreuve vous a semblé suffisante							
6. La consigne de départ était très claire et sans ambiguïté							
7. On a eu l'occasion de demander des précisions.							
8. L'examen a évalué un large éventail de connaissances							
9. L'examen a évalué un large éventail de compétences cliniques							
10. L'examen reflète les exigences de votre future profession							
11. il y avait une adéquation entre les scénarios simulés et les apprentissages cliniques							
12. les scénarios simulés m'ont aidé à intégrer les notions de mon futur métier							
13. L'examen était bien structuré et séquencé							
**Organization de l'ECOS**							
14. L'environnement était calme et propice à l'examen avec peu de distractions							
15. Les équipements comprenant des simulateurs, des instruments médicaux et le matériel nécessaire étaient disponibles et avaient une bonne qualité							
16. Il est facile de terminer au bon moment, pour les différentes stations, le temps imparti était suffisant							
17. Le nombre de stations a été inadéquat							
18. L'examen était bien organisé							
**Caractéristiques de l'ECOS**							
19. Le format de l'examen (ECOS) était plus exempt de stress que le format classique (examen sur terrain).							
20. L'examen était intimidant							
21. L'ECOS vous a semblé très proche, de la réalité des stages,							
22. Par rapport à l'évaluation clinique normative classique, Avant l'évaluation vous étiez plus serein							
23. Les divers modèles structurés(stations) ont contribué à maintenir l'intérêt et l'attention							
24. Le respect de l'asepsie était très facile à réaliser							
25. Le contact avec le patient était très proche par rapport à la réalité							
26. L'ECOS vous a semblé très proche, des évaluations classiques.							
27. L'ECOS vous donne la possibilité de travailler habilement et librement par rapport à l'examen clinique(sur terrain)							
28. L'examen de cette façon donne plus d'occasions de penser et vous amène à réfléchir							
29. L'examen de cette façon révèle les points forts et les points faibles de l'étudiant							
30. L'ECOS donne une rétroaction sur le rendement qui peut être utilisé pour l'auto‐amélioration							
31. La technique évaluée était facile à réaliser							
32. L'ECOS donne moins de chance à l'enseignant de se détacher							
33. L'ECOS réduit au minimum les chances d'échec							
34. L'ECOS permet de compenser le mauvais rendement d'autres stations et/ou d'autres examens							
35. Le format de l'ECOS offre plus d'opportunités que le format conventionnel							
**Validité et fiabilité de l'ECOS**							
36. L'ECOS fournit une véritable mesure des compétences cliniques essentielles							
37. L'ECOS est un examen bien normalisé							
38. Avoir des questions et des scénarios semblables pour tous les élèves est une bonne chose							
39. Ce format d'examen réduit la subjectivité							
40. L'ECOS vous a semblé plus juste, par rapport à l'équité de l'évaluation							
41. La personnalité, l'ethnicité et le sexe n'affecteront pas les scores de l'ECOS							
42. Garder l'utilization des ECOS simulés pour les futures étudiantes							

## References

[1] Majumder MAA , Kumar A , Krishnamurthy K , Ojeh N , Adams OP , Sa B . An evaluative study of objective structured clinical examination (OSCE): students and examiners perspectives. Adv Med Educ Pract [Internet]. 2019;10:387‐397. Accessed April 22, 2023. https://www.tandfonline.com/doi/abs/10.2147/AMEP.S197275 31239801 10.2147/AMEP.S197275 PMC6556562

[2] Schuwirth LWT , van der Vleuten CPM . A history of assessment in medical education. Adv Health Sci Educ. 2020;25(5):1045‐1056. Accessed December 14, 2023. 10.1007/s10459-020-10003-0 33113056

[3] Al‐Haqan A , Al‐Taweel D , Koshy S , Alghanem S . Evolving to objective structured clinical exams (OSCE): transitional experience in an undergraduate pharmacy program in Kuwait. Saudi Pharm J. 2021;29(1):104‐113. Accessed November 17, 2023. https://www.sciencedirect.com/science/article/pii/S1319016420302991 33603545 10.1016/j.jsps.2020.12.013 PMC7873743

[4] Solà‐Pola M , Morin‐Fraile V , Fabrellas‐Padrés N , et al. The usefulness and acceptance of the OSCE in nursing schools. Nurse Educ Pract [Internet]. 2020;43:102736. Accessed November 24, 2023. https://www.sciencedirect.com/science/article/pii/S1471595318303287 32058920 10.1016/j.nepr.2020.102736

[5] Alinier G . Nursing students' and lecturers' perspectives of objective structured clinical examination incorporating simulation. Nurse Educ Today. 2003;23(6):419‐426. Accessed December 14, 2023. https://www.sciencedirect.com/science/article/pii/S0260691703000443 12900190 10.1016/s0260-6917(03)00044-3

[6] Hammad M , Oweis Y , Taha S , Hattar S , Madarati A , Kadim F . Students' opinions and attitudes after performing a dental OSCE for the first time: a Jordanian experience. AADS Proceedings. 2013;77(1):99‐104. 23314473

[7] Terry R , Hing W , Orr R , Milne N . Relationships between pre‐clinical summative assessment scores and the clinical performance of physiotherapy students. J Allied Health. 2020;49(1):13. 32128543

[8] Evans BW , Alinier G , Kostrzewski A , Lefteri KA , Dhillon S Development and design of objective structured clinical examinations (OSCE) in undergraduate pharmacy education in a new School of Pharmacy in England. 2011; Accessed December 14, 2023. http://uhra.herts.ac.uk/handle/2299/9333

[9] Alinier G , Alinier N Design of an objective assessment tool to evaluate students' basic electrical engineering skills: the OSTE. 2006; Accessed December 14, 2023. http://uhra.herts.ac.uk/handle/2299/6140

[10] Kraiem AM , Amri C , El Mhamdi S , Bousriha A , Haj Salem N , Khalfallah T . Evaluation of student's competencies on social Medicine internship with the objective structured clinical examination method. Inte J Educ Res Rev. 2017;2(2):21. Accessed December 14, 2023. https://dergipark.org.tr/en/pub/ijere/issue/30197/327605

[11] Safer M , Zemni I , Mili M , et al. Eating disorders: prevalence and associated factors among health occupation students in Monastir University (Tunisia). Tunis Med. 2020;98(12):895‐912. 33479992

[12] École Supérieure des Sciences et Techniques de la Santé de Sousse . ESSTS Sousse [Internet]. 2023. Accessed November 25, 2023. http://www.esstssousse.tn/public/

[13] Soltani BA , Koubaa AA , Boughalleb W , et al. Internship Competencies in General Medicine (Monastir‐Tunisia). J Gen Pract. 2018;6(3). https://pdfs.semanticscholar.org/5300/ccfd166dcdde110fe14b1de96e6fbd83d375.pdf

[14] Tlili MA , Aouicha W , Sahli J , et al. Prevalence of burnout among health sciences students and determination of its associated factors. Psychol Health Med. 2021;26(2):212‐220. Accessed April 9, 2024. 10.1080/13548506.2020.1802050 32835517

[15] Farhat H , Alinier G , Gangaram P , et al. Exploring pre‐hospital healthcare workers' readiness for chemical, biological, radiological, and nuclear threats in the State of Qatar: a cross‐sectional study. Health Sci Rep [Internet]. 2022;5(5):e803. Accessed September 2, 2022. https://onlinelibrary.wiley.com/doi/abs/10.1002/hsr2.803 36090624 10.1002/hsr2.803 PMC9428763

[16] Farhat H , Alinier G , El Aifa K , et al. Quality improvement tools to manage emergency callbacks from patients with diabetes in a prehospital setting. BMJ Open Qual. 2023;12(1):e002007. 10.1136/bmjoq-2022-002007 PMC9815010 36599502

[17] Aminizadeh M , Rasouli ghahfarokhi SM , Pourvakhshoori N , Beyramijam M , Majidi N , Shahabi Rabori MA . Comparing the effects of two different educational methods on clinical skills of emergency intermediate technician: A quasi‐experimental research. J Educ Health Promot [Internet]. 2019;8:54. Accessed June 25, 2022. https://www.ncbi.nlm.nih.gov/pmc/articles/PMC6442246/ 31008121 10.4103/jehp.jehp_323_18 PMC6442246

[18] Ahmet A , Gamze K , Rustem M , Sezen KA . Is video‐based education an effective method in surgical education? A systematic review. J Surg Educ. 2018;75(5):1150‐1158. Accessed May 4, 2022. https://www.sciencedirect.com/science/article/pii/S1931720417306803 29449162 10.1016/j.jsurg.2018.01.014

[19] Alinier G , Shehatta AL , Makker R . Simulation for Clinical Skills in Healthcare Education. In: Nestel D , Reedy G , McKenna L , Gough S , editors, eds. Clinical Education for the Health Professions: Theory and Practice [Internet. Springer; 2020:[cited 2023 Dec 14] 1‐21. Available from: 10.1007/978-981-13-6106-7_93-1

[20] Farhat H , Laughton J , Joseph A , Abougalala W , Dhiab MB , Alinier G . The educational outcomes of an online pilot workshop in CBRNe emergencies. J Emerg Med Trauma Acute Care. 2022;2022(5):38.

[21] Akhund S , Kadir MM . Do community medicine residency trainees learn through journal club? An experience from a developing country. BMC Med Educ. 2006;6:43. 16925800 10.1186/1472-6920-6-43 PMC1564014

[22] Schwill S , Fahrbach‐Veeser J , Moeltner A , et al. Peers as OSCE assessors for junior medical students – a review of routine use: a mixed methods study. BMC Med Educ. 2020;20(1):17. Accessed November 28, 2023. 10.1186/s12909-019-1898-y 31948425 PMC6966898

[23] Harden RM A Practical Guide for Medical Teachers. 2021; Accessed November 28, 2023. http://thuvienso.thanglong.edu.vn//handle/TLU/6780

[24] Memon S , Shaikh SU . Comparison of performance on written and OSCE assessment during end semester pediatric examination. Pak J Med Sci. 2020;36(4):711‐716. Accessed November 28, 2023. https://www.ncbi.nlm.nih.gov/pmc/articles/PMC7260906/ 32494261 10.12669/pjms.36.4.2026 PMC7260906

[25] Kakadia R , Chen E , Ohyama H . Implementing an online OSCE during the COVID‐19 pandemic. AADS Proceedings. 2021;85(suppl 1):1006‐1008. Accessed November 28, 2023. https://www.ncbi.nlm.nih.gov/pmc/articles/PMC7404760/ 10.1002/jdd.12323 PMC7404760 32666512

[26] Bevan J , Russell B , Marshall B . A new approach to OSCE preparation ‐ PrOSCEs. BMC Med Educ. 2019;19(1):126. Accessed November 28, 2023. 10.1186/s12909-019-1571-5 31046773 PMC6498564

[27] Musa S , Aliyu‐Zubairu R , Haliru L , Andeyansto EA , Dodo A . Experiences with conducting the objective structured clinical examination (OSCE) as a formative tool at the end of paediatric posting in a new medical school in Nigeria. Niger J Paediatr [Internet]. 2019;46(1):9‐14. Accessed November 28, 2023. https://www.ajol.info/index.php/njp/article/view/212026

[28] Alkhateeb NE , Al‐Dabbagh A , Ibrahim M , Al‐Tawil NG . Effect of a formative objective structured clinical examination on the clinical performance of undergraduate medical students in a summative examination: a randomized controlled trial. Indian Pediatr. 2019;56(9):745‐748. Accessed November 28, 2023. 10.1007/s13312-019-1641-0 31638006

[29] Rasku T Community Paramedicine: An integrated care model in a Primary health care setting [Internet]. Tampere University; 2022. Accessed November 30, 2023. https://trepo.tuni.fi/handle/10024/137422

[30] Bell A , Hammer S , Seymour‐Walsh A . The role of educational theory in the future development of paramedicine as a profession: an integrative review. Australas J Paramed. 2021;18:1‐10. 10.33151/ajp.18.941

[31] Hodges AL , Konicki AJ , Talley MH , Bordelon CJ , Holland AC , Galin FS . Competency‐based education in transitioning nurse practitioner students from education into practice. J Am Assoc Nurse Pract. 2019;31(11):675‐682. Accessed November 30, 2023. https://journals.lww.com/jaanp/Fulltext/2019/11000/Competency_based_education_in_transitioning_nurse.11.aspx?casa_token=-cam3DiAyLkAAAAA:D300kKYSJgCFhudqVdJdd65BVjWpXdot0K7vN-CW_EEzz_WGU3kEpNkoJjmwIS2xG-00ruGtzdiqYkS37LNc8A 31584507 10.1097/JXX.0000000000000327

[32] Patel S , Pelletier‐Bui A , Smith S , et al. Curricula for empathy and compassion training in medical education: a systematic review. PLOS ONE [Internet]. 2019;14(8):e0221412. Accessed November 30, 2023. https://journals.plos.org/plosone/article?id=10.1371/journal.pone.0221412 31437225 10.1371/journal.pone.0221412 PMC6705835

[33] Ferrel MN , Ryan JJ . The impact of COVID‐19 on medical education. Cureus. 2020;12(3):e7492. Accessed November 30, 2023. https://www.ncbi.nlm.nih.gov/pmc/articles/PMC7193226/ 32368424 10.7759/cureus.7492 PMC7193226

[34] Zalat MM , Hamed MS , Bolbol SA . The experiences, challenges, and acceptance of e‐learning as a tool for teaching during the COVID‐19 pandemic among university medical staff. PLOS ONE [Internet]. 2021;16(3):e0248758. Accessed November 30, 2023. https://journals.plos.org/plosone/article?id=10.1371/journal.pone.0248758 33770079 10.1371/journal.pone.0248758 PMC7997029

[35] Shehata MH , Kumar AP , Arekat MR , et al. A toolbox for conducting an online OSCE. The Clinical Teacher. 2021;18(3):236‐242. Accessed April 8, 2024. https://asmepublications.onlinelibrary.wiley.com/doi/abs/10.1111/tct.13285 33063427 10.1111/tct.13285

[36] Tseng LP , Hou TH , Huang LP , Ou YK . Effectiveness of applying clinical simulation scenarios and integrating information technology in medical‐surgical nursing and critical nursing courses. BMC Nurs. 2021;20(1):229. 10.1186/s12912-021-00744-7 34781931 PMC8591873

[37] Latjatih NHF , Roslan NS , Jahn Kassim PS , Adam SK . Medical students' perception and satisfaction on peer‐assisted learning in formative OSCE and its effectiveness in improving clinical competencies. Journal of Applied Research in Higher Education. 2021;14(1):171‐179. Accessed November 30, 2023. 10.1108/JARHE-07-2020-0212

